# Exercise and Use of Enhancement Drugs at the Time of the COVID-19 Pandemic: A Multicultural Study on Coping Strategies During Self-Isolation and Related Risks

**DOI:** 10.3389/fpsyt.2021.648501

**Published:** 2021-03-10

**Authors:** Artemisa R. Dores, Irene P. Carvalho, Julius Burkauskas, Pierluigi Simonato, Ilaria De Luca, Roisin Mooney, Konstantinos Ioannidis, M. Ángeles Gómez-Martínez, Zsolt Demetrovics, Krisztina Edina Ábel, Attila Szabo, Hironobu Fujiwara, Mami Shibata, Alejandra Rebeca Melero Ventola, Eva Maria Arroyo-Anlló, Ricardo M. Santos-Labrador, Inga Griskova-Bulanova, Aiste Pranckeviciene, Kei Kobayashi, Giovanni Martinotti, Naomi A. Fineberg, Fernando Barbosa, Ornella Corazza

**Affiliations:** ^1^Laboratory of Neuropsychophysiology, Faculty of Psychology and Education Sciences, University of Porto, Porto, Portugal; ^2^School of Health, Polytechnic of Porto, Porto, Portugal; ^3^Clinical Neurosciences and Mental Health Department and CINTESIS, School of Medicine, Porto, Portugal; ^4^Laboratory of Behavioral Medicine, Neuroscience Institute, Lithuanian University of Health Sciences, Kaunas, Lithuania; ^5^Department of Clinical, Pharmaceutical and Biological Sciences, School of Life and Medical Sciences, University of Hertfordshire, Hatfield, United Kingdom; ^6^Medical Sciences Division, Department of Psychiatry, University of Oxford, Oxford, United Kingdom; ^7^Department of Psychiatry, University of Cambridge, Cambridge, United Kingdom; ^8^Cambridge and Peterborough NHS Foundation Trust, Cambridge, United Kingdom; ^9^Department of Psychology, Pontifical University of Salamanca, Salamanca, Spain; ^10^Institute of Psychology, ELTE Eötvös Loránd University, Budapest, Hungary; ^11^Centre of Excellence in Responsible Gaming, University of Gibraltar, Gibraltar; ^12^Institute of Health Promotion and Sport Sciences, ELTE Eötvös Loránd University, Budapest, Hungary; ^13^Department of Neuropsychiatry, Graduate School of Medicine, University of Kyoto, Kyoto, Japan; ^14^Artificial Intelligence Ethics and Society Team, RIKEN Center for Advanced Intelligence Project, Saitama, Japan; ^15^Faculty of Psychology, Pontifical University of Salamanca, Salamanca, Spain; ^16^Department of Psychobiology, Neuroscience Institute of Castilla-León, University of Salamanca, Salamanca, Spain; ^17^Department of Physical Education, University Teacher's College ‘Fray Luis de León’, Valladolid, Spain; ^18^Department of Neurobiology and Biophysics, Institute of Biosciences, Vilnius University, Vilnius, Lithuania; ^19^Department of Neuroscience, Imaging, and Clinical Science “G. d'Annunzio” University of Chieti-Pescara, Chieti, Italy; ^20^Department of Psychology and Cognitive Science, University of Trento, Trento, Italy

**Keywords:** compulsive exercise, performance-enhancing substances, body image, body dysmorphic disorders, obsessive-compulsive disorder

## Abstract

**Introduction:** Little is known about the impact of restrictive measures during the COVID-19 pandemic on self-image and engagement in exercise and other coping strategies alongside the use of image and performance-enhancing drugs (IPEDs) to boost performance and appearance.

**Objectives:** To assess the role of anxiety about appearance and self-compassion on the practice of physical exercise and use of IPEDs during lockdown.

**Methods:** An international online questionnaire was carried out using the Exercise Addiction Inventory (EAI), the Appearance Anxiety Inventory (AAI), and the Self-Compassion Scale (SCS) in addition to questions on the use of IPEDs.

**Results:** The sample consisted of 3,161 (65% female) adults from Italy (41.1%), Spain (15.7%), the United Kingdom (UK) (12.0%), Lithuania (11.6%), Portugal (10.5%), Japan (5.5%), and Hungary (3.5%). The mean age was 35.05 years (*SD* = 12.10). Overall, 4.3% of the participants were found to engage in excessive or problematic exercise with peaks registered in the UK (11.0%) and Spain (5.4%). The sample reported the use of a wide range of drugs and medicines to boost image and performance (28%) and maintained use during the lockdown, mostly in Hungary (56.6%), Japan (46.8%), and the UK (33.8%), with 6.4% who started to use a new drug. Significant appearance anxiety levels were found across the sample, with 18.1% in Italy, 16.9% in Japan, and 16.7% in Portugal. Logistic regression models revealed a strong association between physical exercise and IPED use. Anxiety about appearance also significantly increased the probability of using IPEDs. However, self-compassion did not significantly predict such behavior. Anxiety about appearance and self-compassion were non-significant predictors associated with engaging in physical exercise.

**Discussion and Conclusion:** This study identified risks of problematic exercising and appearance anxiety among the general population during the COVID-19 lockdown period across all the participating countries with significant gender differences. Such behaviors were positively associated with the unsupervised use of IPEDs, although no interaction between physical exercise and appearance anxiety was observed. Further considerations are needed to explore the impact of socially restrictive measures among vulnerable groups, and the implementation of more targeted responses.

## Introduction

On March 11, 2020, the World Health Organization declared the coronavirus disease 2019 (COVID-19) outbreak to be a pandemic situation as a result of the severe acute respiratory syndrome associated with the coronavirus 2 (SARS-CoV-2) and its highly contagious nature (1). This virus can affect the immune response and, in addition to respiratory complications (2), can have adverse effects on brain function and mental health (3, 4).

Since then, governments, health authorities, and citizens have adopted several measures to combat the spread of the virus (1, 5–7). These included physical distancing (also known as social distancing), prophylactic isolation, mandatory lockdown, and mandatory quarantine (8), leading to altered lifestyles and habits and affecting millions of individuals worldwide (9), society (10, 11), and the economy (12–14). For example, exposure to chronic and daily stressors such as quarantine can affect the cardiovascular system and the emotional experience of the individual, leading to an increased risk of developing a cardiovascular disease or mental illness (15).

Such changes could lead to distress or impairment of citizens' physical, social, and occupational domains (16), generating risk conditions that potentially affect the mental well-being of the general population, especially of those who are most exposed and vulnerable, such as patients diagnosed with COVID-19, those who have been in quarantine or other forms of social isolation, frontline healthcare providers [(6, 17, 18); for a review], and possibly other key workers (i.e., those workers who are crucial to keeping the country running safely, such as police officers, journalists, people delivering food and transportation).

Some of these measures are not new and have been implemented during other outbreaks in the past, such as severe acute respiratory syndrome (SARS), Middle East respiratory syndrome-related coronavirus (MERS), or Ebola (19, 20). However, the global occurrence of this pandemic might intensify the already known effects of both the pandemic and the sanitary control measures on individuals' mental health (21), warranting additional studies (22).

The potentially addictive nature of physical exercise has received increasing interest in the mental health literature (23–30). Social pressure to have a perfect body as a synonym of personal value and success, particularly in Western societies, is transforming the value and meaning attributed to the practice of exercise. Exercise is being increasingly used as a path to boost appearance, rather than primarily as a path to health, or as a pleasurable activity in itself (23, 30). Social media have been contributing to such a “fitspirational” trend, namely, through the continuous posting of photos and videos displaying “perfect bodies,” or inspirational messages encouraging exercising, often beyond the human physical limits (31–34). Such potential damaging content might have an increased effect on adolescents and individuals with mental health problems (35), who might feel unable to meet such unrealistic beauty ideals. Physical exercise can thus become excessive and even problematic, depending also on the way in which people experience their bodies (36, 37).

Excessive and problematic physical exercise, sometimes called “compulsive exercise,” “excessive exercising,” (38) or “exercise addiction” (EA) (24), is a matter of increasing global concern (23).

Brown's (39) and, more recently, Griffith's six components of addiction (25–27) (i.e., salience, mood modification, tolerance, withdrawal symptoms, conflict, relapse) have been used to distinguish EA from other situations in which the individuals are only highly committed to exercising (38). However, as a controversial term, the construct of EA has not been included in the section of behavioral addictions of the main manuals of mental disorders [e.g., the International Classification of Diseases 11th Revision (40) and the Diagnostic and Statistical Manual of Mental Disorders, 5th Edition (DSM-5) (41)], calling for the need of additional sound theory-driven research and clinical evidence that clarify its nature and manifestations.

The relationship between problematic exercise and gender has been inconsistent. Some studies show higher exercise dependence among males (42–44), and others suggest the opposite (45, 46). The association between problematic exercise and age has also been contradictory. Whereas, adulthood has been considered a critical age period for developing problematic exercise in some studies (47), previous studies have reported that the prevalence of exercise dependence should decline with age, or that older adults are less at risk for exercise dependence (42, 48, 49). These differences across studies are possibly explained by methodological issues (e.g., instruments used, sample characteristics comprising mainly college students). Exercise dependence might have changed over time as well, suggesting the need for both longitudinal and current studies with diverse populations (47).

Problematic exercise has been associated with the escalating consumption of image and performance-enhancing drugs (IPEDs) (23), also known as lifestyle drugs, “an umbrella term that encompasses a variety of different products including anabolic steroids, sexual enhancers, growth hormones, and other drugs that can alter the functions of the body to enhance muscle growth, reduce body fat, and promote weight loss” [(50) cit in 29, p. 2]. IPEDs refer to a wide range of products, which are presented as having the potential to improve mental and physical functions. They include drugs for enhancing muscle structure and function (i.e., anabolic drugs), for weight loss, for modifying the aging process, beauty, and cosmetic appearance (i.e., image-enhancing drugs), for improving sex performance [i.e., “sex drugs,” aphrodisiacs, or phosphodiesterase type 5 (PDE5) inhibitors], cognitive performance (i.e., cognitive enhancers), among other functions (23). Online and TV advertisements are contributing to, and exacerbating, the use of these drugs through misleading marketing strategies that promise rapid and safe appearance, physical and mental improvement, and as alternatives to gold standard pharmaceutical products (51–55). However, IPEDs might contain undisclosed ingredients with potential damaging effects to unaware users.

Dissatisfaction with one's own body image and related anxiety about one's appearance might further motivate such a hazardous intake with the purpose of improving physical and mental well-being. In extreme cases, anxiety about appearance might be symptomatic of body dysmorphic disorder (BDD) (23). BDD, classified under the DSM-5's Obsessive–Compulsive and Related Disorders (41), is characterized by extreme dissatisfaction with minor irregularities in one's appearance alongside the irresistible urge to act to eradicate these irregularities. In males, BDD often takes the form of muscle dysmorphia, where the dissatisfaction focuses on aspects of physique that the individual attempts to remediate through the compulsive use of muscle-enhancing agents and physical exercise (23). BDD has been associated with other clinical conditions, including obsessive–compulsive disorder, eating disorders, and addictive behaviors (56, 57). BDD causes considerable distress and interferes significantly with physical and social functioning (41, 58, 59). Yet, it is an under-recognized and underdiagnosed condition (59), namely, because people suffering from it rarely seek intervention for the condition itself, rather for the perceived flaws or for the related mental disorders (e.g., addictive behaviors). Although the specific etiology and pathophysiology of BDD are still under debate, within the spectrum of severe obsessive–compulsive behaviors [e.g., (56)], this is one of the most likely mental disorders to manifest alongside both problematic exercise and the use of IPEDs (23).

In contrast, other types of psychological functioning, if present, might contribute to mitigate or prevent the excessive use of physical exercise and IPEDs. For example, self-compassion is involved in emotional self-regulation and has been associated with psychological benefits among young adults (60). This understanding attitude toward oneself is associated with self-acceptance and self-nurturing abilities and therefore might act as a buffer in a number of mental disorders (61).

Considering the restricted activity associated with the COVID-19 pandemic (e.g., closure of gyms), social distancing might also be expected to be beneficial as much as by reducing several of the most frequent everyday stressors (62), individuals may be induced to relax their exercise habits and compulsive need for IPEDs and the anxiety about body image may be reduced. However, to date, young adults have rated everyday events as more intensely stressful during physical isolation (62). Therefore, the lack of physical contact with support networks might conversely trigger additional mental health problems as a result of the quarantine (16, 17, 21). Prolonged exposure to TV and online information and advertisements during confinement might have also had an impact on people's mood, image, performance, physical exercise, and IPEDs consumption.

In this work, we investigate the impact of the socially restrictive measures imposed by the COVID-19 pandemic on self-image and the practice of excessive, or even potentially problematic, physical exercise and the use of IPEDs as coping strategies to boost appearance during the period of its most restrictive policies (April–May 2020). We also consider the role of self-compassion as a potential mitigating factor for such risky behaviors. Considering the unprecedented situation, we hypothesized that individuals might have engaged more compulsively with exercise and IPEDs intake to better cope with the pandemic's altered lifestyle, closure of fitness centers, and reiterated period of self-isolation (63, 64), mainly when self-directed negative feelings, such as anxiety about one's own body, came into play.

The hypothesis is partly based on the results of a pre-COVID-19 investigation where authors found a strong association between “exercise addiction,” IPEDs use, including illicit drugs, and BDD among a large international cohort of regular exercisers (23). Evidence was supported by another more recent study on “exercise addiction” during the COVID-19 pandemic among a Spanish-speaking sporting population (65). Although the overall practice of exercise decreased by almost 50% during the pandemic, the perceived impact of the pandemic on regular exercising did not differ among the three exercise groups (asymptomatic, symptomatic, and at-risk for addiction). The risk of “exercise addiction” was found in ~15% of the sample. As both these studies were carried out among a population of physically active individuals, who exercise on a regular basis, one might wonder about the behaviors across the general population under such extraordinary circumstances. While some individuals were prohibited from practicing their regular physical exercise/activity outdoors (12) and might have stopped their exercise practices, others might have implemented new (unsupervised) workout regimes indoors (66).

More specifically, in this work, we sought: (1) to characterize the practice of physical exercise, (2) to explore the use of IPEDs, and (3) any potential associations between these risky behaviors and self-directed negative feelings of appearance anxiety vs. the positive feelings of self-compassion, along with gender, age, occupation (e.g., key workers) during the start of the COVID-19 pandemic. Results were compared cross-culturally in the United Kingdom (UK), Italy, Hungary, Portugal, Spain, Lithuania, and Japan.

## Materials and Methods

### Study Design

This is an international cross-sectional observational study.

### Participants

The sample comprised 3,161 participants from seven countries: Italy (*n* = 1,300; 41.1%), Spain (*n* = 497; 15.7%), UK (*n* = 378; 12.0%), Lithuania (*n* = 367; 11.6%), Portugal (*n* = 332; 10.5%), Japan (*n* = 175; 5.5%), and Hungary (*n* = 112; 3.5%). Table 1 shows the sociodemographic characteristics of the participants in total and by country. The age of the participants ranged from 15 to 80 years old (*M* = 35.05; *SD* = 12.10), and the majority was female (*n* = 2,046; 65.2%). Most of the sample were highly educated with a master's (*n* = 995; 31.6%), PhD (*n* = 196; 6.2%), or a bachelor's degree (*n* = 951; 30.2%) and employed (*n* = 1,749; 55.7%) or studying (*n* = 666; 21.2%).

**Table 1:** Sociodemographic characteristics of the sample and types of physical activities (*N* = 3,161).

	**Total *N* = 3,161**	**UK *N* = 378**	**Italy *N* = 1,300**	**Spain *N* = 497**	**Hungary *N* = 112**	**Portugal *N* = 332**	**Japan *N* = 175**	**Lithuania *N* = 367**
**Age, years**								
Mean	35.05	36.70	31.89	38.58	41.79	34.24	39.28	36.36
(SD)	(12.10)	(12.24)	(10.96)	(13.56)	(11.80)	(11.18)	(13.84)	(10.33)
Min–Max	15–80	15–75	16–80	15–72	18–66	18–65	18–80	16–66
Gender, Female	2,046	188	857	302	69	249	70	311
(*n*; %)	65.2%	50.7%	66.1%	61.6%	61.6%	76.4%	40.2%	84.7%
**Education level (*****n*****;** ***%*****)**								
PhD	196 (6.2%)	44 (11.7%)	27 (2.1%)	25 (5.1%)	3 (2.7%)	22 (6.7%)	17 (9.7%)	58 (15.8%)
Master's degree	995 (31.6%)	140 (37.3%)	418 (32.2%)	81 (16.4%)	41 (36.6%)	97 (29.4%)	21 (12.0%)	197 (53.7%)
Bachelor's degree	951 (30.2%)	118 (31.5%)	262 (20.2%)	240 (48.7%)	24 (21.4%)	152 (46.1%)	89 (50.9%)	66 18.0%
High school	760 (24.1%)	50 (13.3%)	520 (40.0%)	32 (6.5%)	31 (27.7%)	55 (16.7%)	36 (20.6%)	36 (9.8%)
Other	249 (7.9%)	23 (6.1%)	72 (5.5%)	115 (23.3%)	13 (11.6%)	4 (1.2%)	12 (6.9%)	10 (2.7%)
**Occupation (*****n*****;** ***%*****)**								
Employed	1,749 (55.7%)	231 (61.4%)	659 (51.0%)	248 (50.3%)	74 (66.1%)	136 (41.3%)	122 (70.1%)	279 (76.6%)
Student	666 (21.2%)	60 (16.0%)	332 (25.7%)	98 (19.9%)	9 (8.0%)	88 (26.7%)	37 (21.3%)	42 (11.5%)
Unemployed	244 (7.8%)	13 (3.5%)	132 (10.2%)	53 (10.8%)	6 (5.4%)	25 (7.6%)	5 (2.9%)	10 (2.7%)
Retired	200 (6.4%)	9 (2.4%)	153 (11.8%)	25 (5.1%)	4 (3.6%)	7 (2.1%)	0 (0%)	2 (0.5%)
Freelance/individual activity	282 (9.0%)	63 (16.8%)	17 (1.3%)	69 (14.0%)	19 (17.0%)	73 (22.2%)	10 (5.7%)	31 (8.5%)
Key worker (*n*; %)	1,102 (34.9%)	103 (27.4%)	392 (30.2%)	170 (34.2%)	48 (42.9%)	111 (33.6%)	98 (56.0%)	180 (49.0%)
Health care and related specialities	517 (16.4%)	52 (13.8%)	186 (14.3%)	69 (13.9%)	9 (8.0%)	65 (19.7%)	57 (32.6%)	79 (21.5%)
Teachers and tutors	90 (2.9%)	8 (2.1%)	15 (1.2%)	5 (1.0%)	10 (8.9%)	8 (2.1%)	20 (11.4%)	25 (6.8%)
Transportation	23 (0.7%)	3 (0.8%)	5 (0.4%)	4 (0.8%)	5 (4.5%)	2 (0.6%)	2 (1.1%)	2 (0.5%)
Food industry	69 (2.2%)	7 (1.9%)	20 (1.5%)	24 (4.8%)	3 (2.7%)	1 (0.3%)	2 (1.1%)	12 (3.3%)
Public sector	32 (1.0%)	4 (1.1%)	2 (0.2%)	12 (2.4%)	1 (0.9%)	4 (1.2%)	3 (1.7%)	6 (1.6%)
Government	59 (1.9%)	6 (1.6%)	22 (1.7%)	8 (1.6%)	1 (0.9%)	5 (1.5%)	2 (1.1%)	15 (4.1%)
Postal and other services	34 (1.1%)	1 (0.3%)	16 (1.2%)	13 (2.6%)	3 (2.7%)	0 (0%)	0 (0%)	1 (0.3%)
National or public security	40 (1.3%)	7 (1.9%)	9 (0.7%)	10 (2.0%)	1 (0.9%)	2 (0.6%)	4 (2.3%)	7 (1.9%)
Pharmacy and related activity	23 (0.7%)	3 (0.8%)	11 (0.8%)	4 (0.8%)	1 (0.9%)	2 (0.6%)	1 (0.6%)	1 (0.3%)
Other	187 (5.9%)	23 (6.1%)	71 (5.5%)	0 (0%)	19 (17.0%)	71 (5.5%)	6 (3.4%)	50 (13.6%)
Professional athlete	52 (1.6%)	6 (1.6%)	28 (2.2%)	3 (0.6%)	3 (2.7%)	5 (1.5%)	0 (0%)	7 (1.9%)
Mental disorder (before)	547 (17.4%)	76 (20.2%)	257 (19.9%)	89 (17.9%)	12 (10.7%)	67 (20.3%)	14 (8.0%)	32 (8.8%)
Anxiety	329 (10.5%)	58 (15.4%)	128 (9.9%)	71 (14.3%)	7 (6.3%)	39 (11.8%)	4 (2.3%)	22 (6.0%)
Depression	152 (4.8%)	44 (11.7%)	54 (4.2%)	19 (3.8%)	6 (5.4%)	13 (3.9%)	1 (0.6%)	15 (4.1%)
Other mood disorders	52 (1.7%)	7 (1.9%)	21 (1.6%)	11 (2.2%)	2 (1.8%)	7 (2.1%)	3 (1.7%)	1 (0.3%)
Psychotic disorders	7 (0.2%)	5 (1.3%)	0 (0%)	0 (0%)	0 (0%)	1 (0.3%)	0 (0%)	1 (0.3%)
Eating disorders	73 (2.3%)	16 (4.3%)	30 (2.3%)	14 (2.8%)	2 (1.8%)	3 (0.9%)	1 (0.6%)	7 (1.9%)
Personality disorders	15 (0.5%)	6 (1.6%)	6 (0.5%)	2 (0.4%)	0 (0%)	0 (0%)	0 (0%)	1 (0.3%)
Other(s)	87 (2.8%)	10 (2.7%)	37 (2.9%)	10 (2.0%)	6 (1.8%)	2 (1.8%)	6 (3.4%)	16 (4.4%)
Physical distancing worsened mental disorder	205 (47.9%)	26 (44.8%)	97 (45.1%)	27 (47.4%)	3 (33.3%)	32 (64.0%)	5 (55.6%)	15 (50.0%)
**Physical exercise**								
Generic workout	1,018 (33.1%)	112 (29.9%)	442 (35.0%)	145 (29.7%)	27 (24.3%)	124 (38.5%)	15 (9.2%)	153 (42.7%)
Running	422 (13.7%)	69 (18.4%)	165 (13.1%)	66 (13.5%)	31 (27.9%)	33 (10.2%)	21 (12.9%)	37 (10.3%)
Walking	416 (13.5%)	42 (11.2%)	133 (10.5%)	122 (25.0%)	6 (5.4%)	48 (14.9%)	15 (9.2%)	50 (14.0%)
Fighting sports	412 (13.4%)	188 (50.3%)	85 (6.7%)	33 (6.8%)	2 (1.8%)	8 (2.5%)	84 (51.5%)	12 (3.4%)
Martial arts	355 (11.5%)	181 (48.4%)	42 (3.3%)	30 (6.1%)	1 (0.9%)	6 (1.9%)	83 (50.9%)	12 (3.4%)
Yoga	306 (9.9%)	47 (12.6%)	110 (8.7%)	43 (8.8%)	15 (13.5%)	31 (9.6%)	14 (8.6%)	46 (12.8%)
Swimming	216 (7.0%)	26 (7.0%)	90 (7.1%)	24 (4.9%)	32 (28.8%)	17 (5.3%)	8 (4.9%)	19 (5.3%)
Weight lifting	210 (6.8%)	32 (8.6%)	99 (7.8%)	10 (2.0%)	12 (10.8%)	26 (8.1%)	5 (3.1%)	26 (7.3%)
Cycling	172 (5.6%)	28 (7.5%)	64 (5.1%)	22 (4.5%)	18 (16.2%)	13 (4.0%)	4 (2.5%)	23 (6.4%)
Ball sports	160 (5.2%)	19 (5.1%)	79 (6.3%)	27 (5.5%)	8 (7.2%)	17 (5.3%)	3 (1.8%)	7 (2.0%)
Other	129 (4.2%)	32 (8.6%)	48 (3.8%)	10 (2.0%)	8 (7.2%)	7 (2.2%)	7 (4.3%)	17 (4.7%)
Dance	118 (3.8%)	15 (4.0%)	49 (3.9%)	17 (3.5%)	6 (5.4%)	9 (2.8%)	2 (1.2%)	20 (5.6%)
Mountaineering	85 (3.1%)	15 (4.0%)	38 (3.0%)	20 (4.1%)	6 (5.4%)	-	1 (0.6%)	5 (1.4%)
Cross fit	82 (2.7%)	8 (2.1%)	32 (2.5%)	16 (3.3%)	3 (2.7%)	17 (5.3%)	0 (0%)	6 (1.7%)
Tennis	57 (1.9%)	7 (1.9%)	8 (0.6%)	19 (3.9%)	6 (5.4%)	4 (1.2%)	4 (2.5%)	9 (2.5%)
Triathlon	30 (1.0%)	1 (0.3%)	25 (2.0%)	0 (0%)	2 (1.8%)	1 (0.3%)	0 (0%)	1 (0.3%)
No activity	452 (14.7%)	11 (2.9%)	239 (18.9%)	67 (13.7%)	2 (1.8%)	51 (15.8%)	13 (8.0%)	69 (19.3%)

*Note: The percentages do not add up to 100 because some people reported more sports they use and more than one key worker job*.

A considerable number of participants were “key workers” (*n* = 1,106; 35.0%), most of them in health professions (*n* = 517; 16.4%).

A total of 564 participants reported mental health problems before the pandemic (17.4%). Anxiety was the most prevalent of the reported mental problems (*n* = 329; 10.5%), followed by depression (*n* = 152; 4.8%). Almost half of the participants who reported the presence of a mental disorder before the pandemic considered that the physical distancing has worsened their mental problem (*n* = 205; 47.9%).

Most of the participants engaged in fitness activities, mainly generic workouts (*n* = 1,018; 33.1%), running (*n* = 422; 13.7%), walking (*n* = 416; 13.5%), fight sports (e.g., boxing, kickboxing, judo, sumo, and karate) (*n* = 412; 13.4%), and martial arts (e.g., aikido, Brazilian Jiu-Jitsu, Krav Maga, Kung Fu) (*n* = 355; 11.5%). A small proportion of the respondents did not practice physical exercise (*n* = 422; 14.7%).

### Procedure

The study was approved by the Human Sciences Ethics Committee at the University of Hertfordshire (HSK/SF/UH/00104) and by the Ethics Committees of each participating country. It complied with the Declaration of Helsinki and with the European General Data Protection Regulation. The study's presentation included the project's description and aims, followed by an informed consent form. Upon agreement to participate, a link to the questionnaire was sent to participants. The latter was translated and back-translated from English into different languages (Italian, Spanish, Japanese, Portuguese, Hungarian, Lithuanian). Data collection was implemented *via* the Web-based survey platform Qualtrics [Qualtrics, Provo, UT, 2020], and the data were stored in a secure platform at the University of Hertfordshire.

Recruitment was supported by an already established global network of collaborators in Italy, UK, Lithuania, Hungary, Portugal, Spain, and Japan. It mainly occurred *via* posts on health and well-being social media platforms, not necessarily fitness related, namely, Facebook, LinkedIn, WhatsApp, Twitter, and Instagram. A snowball sampling technique was used; participants were invited to complete the survey and share it with their contacts. These procedures ensured a heterogeneous sample inclusive of both sporting and non-sporting populations.

Data collection occurred during April and May 2020, precisely at the peak of the COVID-19 pandemic and coinciding with the lockdown period in all the participating countries.

### Instruments

The questionnaire was composed of: (a) sociodemographic information; (b) the Exercise Addiction Inventory (EAI); (c) questions on the use of IPEDs (i.e., “Have you taken supplements/products to reach your fitness goal/physical appearance during self-isolation? [Choose yes or no]”; “What are they? [Tick as many as apply]”); (d) the Appearance Anxiety Inventory (AAI); and (e) the Self-Compassion Scale (SCS).

The EAI-brief (67, 68) was developed to assess the level of engagement in physical activity. The EAI-brief is based on a modified version of the components of behavioral addiction by Griffiths (24) and consists of six questions that reflect the six general components of addiction (i.e., salience, mood modification, tolerance, withdrawal symptoms, social conflict, and relapse). Participants rate their responses on a 5-point Likert scale ranging from 1 (strongly disagree) to 5 (strongly agree). A sum score is calculated (for a maximum of 30 points), with higher scores indicating the presence of more problems. A score equal to 24 or higher indicates problematic exercise akin to addiction. This cutoff represents those individuals with scores in the top 15% of the total scale score in the original study. The EAI is a theoretically driven instrument with valid and reliable psychometric properties reported in several studies across many countries (68–70). In our sample, Cronbach's *alpha* was 0.72, ranging from 0.65 to 0.75 for the different countries.

The intake of a wide range of IPEDs was assessed with questions developed for the purposes of this study. For each question, respondents answered “yes” or “no” or selected the response from a list of options. For purposes of comparison, listed products included all those used in a previous study by Corazza et al. (23), developed after consultation with experts, namely, sport nutritionists and clinicians.

The AAI (71) measures the cognitive and behavioral dimensions of anxiety about body image in general and provides an indication of symptoms associated with BDD. It is a 10-item self-report questionnaire that assesses the frequency of avoidance behavior and of monitoring threats (e.g., checking, self-focused attention) that are characteristic of responses to a distorted body image. In its original version, each item is rated on a 5-point Likert scale ranging from 0 (not at all) to 4 (all the time), yielding a summary score with a maximum of 40 points. Higher scores indicate a higher occurrence of appearance anxiety. It has been used to assess change in psychotherapy with patients suffering from BDD. In our version, the AAI included a four-point Likert scale ranging from 1 (not at all) to 4 (all the time), for a maximum of 40 points. The cutoff score for this version was defined using the same methodology as for the EAI questionnaire, i.e., values ≥21 based on the scores falling in the top 15% of the total scale score. In our sample, Cronbach's *alpha* was 0.87, ranging from 0.81 to 0.90 for the different countries in this study.

The SCS-Short Form (72) consists of compassion turned to oneself and is related to emotional self-regulation. It consists of 12 items distributed by six subscales: Self-Kindness, Self-Judgment, Common Humanity, Isolation, Mindfulness, and Over-Identification. Respondents are asked to answer each item on a 5-point scale ranging from 1 (almost never) to 5 (almost always) according to “how I typically act toward myself in difficult times.” The total score of the SCS (maximum of 60 points) is computed through the sum of the scores on the six subscales (with some of them being reversed previously). Higher scores indicate greater self-compassion. The SCS lacks an official cutoff score. Consistent with the procedures for the AAI, we used the cutoff score <27 to represent those 15% of the study group who were the least self-compassionate. In our sample, Cronbach's *alpha* was 0.82, ranging from 0.80 to 0.84 for the different countries in this study.

### Data Analysis

Normality checking yielded adequate values, and SPSS for Windows, version 17.0 (SPSS Inc., Chicago, Illinois), was used for all analyses.

Descriptive analyses (frequency, central tendency, and dispersion measures) were used for the following variables: sociodemographic characteristics (age, gender, occupation), use of IPEDs and sources where the IPEDs were obtained, the EAI, the AAI, and the SCS. Student's *t*-tests were calculated to compare means on the EAI, AAI, and SCS between male and female participants. Chi-square tests were used for categorical variables, to compare scores (e.g., above/below the cutoff point for each instrument) between male and female participants, and by country.

Binary logistic regressions were calculated to inspect (1) how AAI, SCS, and IPEDs use predict the practice of physical exercise (classified as 0, “no practice,” or 1, “practice”), controlling for age, and (2) how AAI, SCS, and EAI predict IPEDs consumption (classified as 0, “not used,” or 1, “used”), also controlling for age. These same logistic regression models were then run for each gender. In addition, (3) logistic regressions were conducted to inspect reported changes in physical exercise, in IPEDs consumption, and in mental health state during the lockdown (as outcome variables) and the predictors of such changes. A new predictor was entered in these latter models [namely, whether or not respondents were key workers (0, “non-key worker”; 1, “key worker”)] in addition to the other variables mentioned above [AAI, SCS, IPEDs use, EAI, gender (0, men; 1, women), and age] for inspection of the role of key workers in these changes. Professional athletes represented a very small proportion of the total sample and were removed from the first regression analysis so that results better reflect the population at large. With a given sample size allowing *R*^2^ for a 2% change and the number of predictor variables ranging from 4 to 7, we were able to achieve power ranging from 0.74 to 0.99.

## Results

### Physical Exercise

Among 3,161 participants from seven countries included in this study, results showed a mean score of 16.63 (*SD* = 4.32) on the EAI, with male participants displaying significantly higher values (*M* = 16.99; *SD* = 4.41) than their female counterparts (*M* = 16.43; *SD* = 4.25), *t*_(2946)_ = 3.31; *p* = 0.001; *d* = 0.13. Scores equal to or above the cutoff point of 24, indicating problematic exercise akin to addiction, were observed among 4.3% (*n* = 128) of the total sample. This group of high scorers also included a significantly greater proportion of male (*n* = 60; 5.9%) than female participants (*n* = 66; 3.4%), χ^2^ (1, *N* = 2,946) = 9.58, *p* = 0.002; *N* = 126. In addition, major cross-cultural differences were found in the comparison among those scoring above/below the cutoff point of 24 across the participating countries (Table 2). Those at risk of more problematic forms of exercise were mainly found in the UK (11%) and Spain (5.4%).

**Table 2:** Problematic exercise (EAI ≥24): total and by country.

	**Total *n* (*%*)**	**UK *n* (*%*)** ***N* = 363**	**Italy *n* (*%*)** ***N* = 1,211**	**Spain *n* (*%*)** ***N* = 463**	**Hungary *n* (*%*)** ***N* = 107**	**Portugal *n* (*%*)** ***N* = 316**	**Japan *n* (*%*)** ***N* = 172**	**Lithuania *n* (*%*)** ***N* = 337**	**Country differences**
**EAI**	128	40	37	25	4	9	5	8	χ^2^ = 51.17
**(scores ≥ 24)**	(4.3%)	(11.0%)	(3.1%)	(5.4%)	(3.7%)	(2.8%)	(2.9%)	(2.4%)	*p* < 0.001
*n* = 2,969									

### Use of Image and Performance-Enhancing Drugs (IPEDs)

Just over a quarter of participants (28%, *N* = 2,684) had used IPEDs (Table 3). Among them, 19.7% reported using IPEDs before the restrictive measures and maintaining this behavior during the lockdown; only 1.5% had stopped consuming IPEDs (Table 3). In addition, 6.4% of the total sample started consuming IPEDs during this period.

**Table 3:** Use of fitness supplements (IPEDs): total and by country.

	***N* 2,684**	**UK *n* (*%*)**	**Italy *n* (*%*)**	**Spain *n* (*%*)**	**Hungary *n* (*%*)**	**Portugal *n* (*%*)**	**Japan *n* (*%*)**	**Lithuania *n* (*%*)**	**Country differences**
Have never used	1,945 (72.5%)	219 (66.2%)	822 (76.2%)	380 (85.8%)	34 (44.2%)	197 (69.1%)	88 (55.0%)	205 (66.3%)	
Have used	739 (28.0%)	112 (33.8%)	257 (24.3%)	63 (14.4%)	43 (56.6%)	88 (31.5%)	72 (46.8%)	104 (34.4%)	χ2 = 118.47 *p < 0.001*
Used before and during isolation	528 (19.7%)	106 (32.0%)	150 (13.9%)	38 (8.6%)	41 (53.2%)	50 (17.5%)	66 (41.3%)	77 (24.9%)	
Used before isolation, have not used during isolation	39 (1.5%)	2 (0.6%)	14 (1.3%)	7 (1.6%)	2 (2.6%)	4 (1.4%)	2 (1.3%)	8 (2.6%)	
Started using during isolation	172 (6.4%)	4 (1.2%)	93 (8.6%)	18 (4.1%)	0 (0.0%)	34 (11.9%)	4 (2.5%)	19 (6.1%)	

*IPEDs, Image and performance-enhancing drugs*.

Major differences emerging from the cross-cultural comparison are displayed in Table 3. Hungary presented the largest percentage of participants who reported using IPEDs (56.6%), followed by Japan (46.8%), then Lithuania (34.4%), the UK (33.8%), and Portugal (31.5%).

Those who started the consumption of IPEDs during self-isolation were mainly from Portugal (11.9%), while those who were already consuming such products and continued during lockdown were mainly from Hungary (53.2%), Japan (41.3%), and the UK (32.0%) (Table 3). A gender difference was found among those who were already using IPEDs before isolation and continued consuming during isolation, χ^2^ (2, *N* = 2,618) = 40.41, *p* < 0.001; *N* = 525, with a greater proportion of male (*n* = 241, 26.8%) than female participants (*n* = 284; 16.5%) reporting continued use.

Across the overall sample, the products that were most frequently used with the purpose of enhancing image and performance were vitamins (40.5%), proteins (40.4%), caffeine (36.2%), tea or infusions (35.7%), multivitamin supplements (33.6%), and amino acids (27.8%), along with other substances such as ibuprofen (10.3%) and antioxidants (8.3%). Participants also reported consumption of stimulants, nitric oxide, beta blockers, and ketones, used by around 2.0%, androgenic substances, namely, steroids and hormones or hormone-related products (each used by 1.4% of the sample), and other products that were reported in smaller percentages (Table 4). These products were purchased mostly in pharmacies (43.8%), followed by the Internet (43.2%), and food stores and specialized food stores (24.9 and 21.8%, respectively). The category “others” was chosen by 4.6% of the respondents, and 0.8% made a purchase from the black market (Table 4). Lithuanians had the highest rates of vitamins, omega 3, and fish oil use as well as positive attitude toward herbal medicine and herbal infusions; the highest prevalence of ibuprofen use was also observed in Lithuania. Participants in Lithuania acquired IPEDs from pharmacies in a very large percentage and larger than respondents from all other countries.

**Table 4:** Use of fitness supplements: type and source of purchase (total and by country).

**Type of fitness product (*n* = 785)**	***n* (%)**	**UK *n* (%)**	**Italy *n* (%)**	**Spain *n* (%)**	**Hungary *n* (%)**	**Portugal *n* (%)**	**Japan *n* (%)**	**Lithuania *n* (%)**
Vitamins	318 (40.5%)	61 (54.5%)	91 (32.9%)	14 (20.3%)	25 (56.8%)	30 (31.9%)	23 (29.5%)	74 (66.7%)
Proteins	317 (40.4%)	70 (62.5%)	105 (37.9%)	27 (39.1%)	14 (31.8%)	43 (45.7%)	29 (37.2%)	29 (26.1%)
Caffeine	284 (36.2%)	41 (36.6%)	91 (32.9%)	22 (31.9%)	16 (36.4%)	42 (44.7%)	27 (34.6%)	45 (40.5%)
Tea or infusions	280 (35.7%)	34 (30.4%)	106 (38.3%)	22 (31.9%)	13 (29.5%)	35 (37.2%)	26 (16.3%)	44 (39.6%)
Multivitamin supplement	264 (33.6%)	39 (34.8%)	103 (37.2%)	12 (17.4%)	20 (45.5%)	25 (26.6%)	19 (24.4%)	46 (41.4%)
Amino acids	218 (27.8%)	33 (29.5%)	87 (31.4%)	16 (23.2%)	11 (25.0%)	17 (18.1%)	30 (38.5%)	24 (21.6%)
Omega 3 fish oil	208 (26.5%)	42 (37.5%)	51 (18.4%)	9 (13.0%)	12 (27.3%)	26 (27.7%)	10 (12.8%)	58 (52.3%)
Multimineral supplement	172 (21.9%)	19 (17.70)	91 (32.9%)	5 (7.2%)	7 (15.9%)	14 (14.9%)	9 (11.5%)	27 (24.3%)
Creatine	152 (19.4%)	35 (31.3%)	52 (18.8%)	19 (27.5%)	3 (6.8%)	21 (22.3%)	7 (9.0%)	15 (13.5%)
Carnitine	98 (12.5%)	8 (7.1%)	34 (12.3%)	12 (17.4%)	5 (11.4%)	19 (20.2%)	3 (3.8%)	17 (15.3%)
Mineral salt	96 (12.2%)	6 (5.4%)	66 (23.8%)	4 (5.8%)	4 (9.1%)	6 (6.4%)	3 (3.8%)	7 (6.3%)
Turmeric	93 (11.8%)	19 (17.0%)	27 (9.7%)	7 (10.1%)	5 (11.4%)	8 (8.5%)	7 (9.0%)	20 (18.0%)
Fish oil	89 (11.3%)	17 (15.2%)	12 (4.3%)	2 (2.9%)	6 (13.6%)	8 (8.5%)	7 (9.0%)	37 (33.3%)
Herbal medicine	88 (11.2%)	13 (11.6%)	24 (8.7%)	9 (13.0%)	7 (15.9%)	4 (4.3%)	6 (7.7%)	25 (22.5%)
Green tea extract	79 (10.1%)	13 (11.6%)	23 (8.3%)	10 (14.5%)	8 (18.2%)	14 (14.9%)	3 (3.8%)	8 (7.2%)
Ibuprofen	71 (10.3%)	20 (17.9%)	1 (0.4%)	9 (13.0%)	1 (2.3%)	0 (0.0%)	8 (10.3%)	32 (28.8%)
Antioxidants	65 (8.3%)	9 (8.0%)	23 (8.3%)	0 (0.0%)	7 (15.9%)	9 (9.6%)	5 (6.4%)	12 (10.8%)
Glutamate	64 (8.2%)	10 (8.9%)	19 (6.9%)	7 (10.1%)	5 (11.4%)	10 (8.9%)	5 (6.4%)	8 (7.2%)
Glucosamine	52 (6.6%)	17 (15.2%)	10 (3.6%)	0 (0.0%)	8 (18.2%)	5 (5.3%)	3 (3.8%)	9 (8.1%)
Taurine	51 (6.5%)	4 (3.6%)	18 (6.5%)	6 (8.7%)	1 (2.3%)	10 (10.6%)	5 (6.4%)	7 (6.3%)
Diuretics	49 (6.2%)	3 (2.7%)	17 (6.1%)	9 (13.0%)	1 (2.3%)	17 (18.1%)	1 (1.3%)	1 (0.9%)
Ginseng	48 (6.1%)	8 (7.1%)	17 (6.1%)	2 (2.9%)	4 (9.1%)	8 (8.5%)	3 (3.8%)	6 (5.4%)
Laxatives	43 (5.5%)	7 (6.3%)	11 (4.0%)	4 (5.8%)	1 (2.3%)	6 (6.4%)	9 (11.5%)	5 (4.5%)
Guaran	38 (4.8%)	7 (6.3%)	11 (4.0%)	2 (2.9%)	1 (2.3%)	12 (12.8%)	1 (1.3%)	4 (3.6%)
Beta alanine	25 (3.2%)	4 (3.6%)	9 (3.2%)	5 (7.2%)	1 (2.3%)	2 (2.1%)	1 (1.3%)	3 (2.7%)
Other^*^	24 (3.2%)	9 (8.0%)	0 (0.0%)	1 (1.4%)	–	6 (6.4%)	6 (7.7%)	2 (1.8%)
Nitric oxide	16 (2.0%)	2 (1.8%)	5 (1.8%)	3 (4.3%)	1 (2.3%)	2 (2.1%)	2 (2.6%)	1 (0.9%)
Stimulants (e.g., amphetamine, modafinil)	15 (1.9%)	5 (4.5%)	1 (0.4%)	0 (0.0%)	1 (2.3%)	5 (4.5%)	1 (1.3%)	2 (1.8%)
Ketones	14 (1.8%)	7 (6.3%)	2 (0.7%)	0 (0.0%)	1 (2.3%)	2 (2.1%)	1 (1.3%)	1 (0.9%)
Beta blockers	13 (1.7%)	1 (0.9%)	1 (0.4%)	0 (0.0%)	3 (6.8%)	3 (3.2%)	2 (2.6%)	3 (2.7%)
Androgenic substances (e.g., steroids)	11 (1.4%)	3 (2.7%)	0 (0.0%)	1 (1.4%)	1 (2.3%)	4 (4.3%)	1 (1.3%)	1 (0.9%)
Hormones (e.g., EPO, insulin) or related (e.g., beta-2 agonists)	11 (1.4%)	2 (1.8%)	1 (0.4%)	0 (0.0%)	1 (2.3%)	3 (3.2%)	2 (2.6%)	2 (1.8%)
Pyruvate	8 (1.0%)	0 (0.0%)	0 (0.0%)	0 (0.0%)	4 (9.1%)	1 (1.1%)	2 (2.6%)	1 (0.9%)
Orlistat	8 (1.0%)	0 (0.0%)	0 (0.0%)	1 (1.4%)	1 (2.3%)	3 (3.2%)	2 (2.6%)	1 (0.9%)
Glucocorticoids	4 (0.5%)	0 (0.0%)	0 (0.0%)	0 (0.0%)	1 (2.3%)	1 (1.1%)	1 (1.3%)	1 (0.9%)
**Source of purchase**	**Total** ***n*** **(%)**	**UK** ***n*** **(%)**	**Italy** ***n*** **(%)**	**Spain** ***n*** **(%)**	**Hungary** ***n*** **(%)**	**Portugal** ***n*** **(%)**	**Japan** ***n*** **(%)**	**Lithuania** ***n*** **(%)**
Pharmacy	313 (43.8%)	40 (38.8%)	101 (40.2%)	17 (29.3%)	22 (52.4%)	24 (28.9%)	38 (52.1%)	71 (67.6%)
Internet	309 (43.2%)	51 (49.5%)	113 (45.0%)	16 (27.6%)	22 (52.4%)	30 (36.1%)	34 (46.6%)	43 (41.0%)
Specialized food store	178 (24.9%)	33 (32.0%)	58 (23.1%)	23 (39.7%)	12 (28.6%)	30 (36.1%)	6 (8.2%)	16 (15.2%)
Food store	156 (21.8%)	33 (32.0%)	47 (18.7%)	16 (27.6%)	5 (11.9%)	26 (31.3%)	17 (23.3%)	12 (11.4%)
Other	33 (4.6%)	4 (3.9%)	15 (6.0%)	3 (5.2%)	3 (7.1%)	2 (2.4%)	3 (4.1%)	3 (2.9%)
Black market	6 (0.8%)	2 (1.9%)	1 (0.4%)	0 (0.0%)	0 (0.0%)	2 (2.4%)	0 (0.0%)	1 (1.0%)

*Note: Selected from multiple choice*;

* *Main answers to the option “Other” products: berberine; black garlic; casein; Chlorella; collagen; collagen UC2; collagen peptides; hydration sport drinks; pea protein isolate. The percentages do not add up to 100 because some people reported more forms of supplements they use*.

### Appearance Anxiety vs. Self-Compassion

Regarding anxiety about one's appearance, the sample's mean value on the AAI was 15.82 (*SD* = 5.11). Female participants (*M* = 16.62; *SD* = 5.29) scored significantly higher than male participants (*M* = 14.31; *SD* = 4.36), *t*_(2872)_ = −11.85; *p* < 0.001; *d* = 0.48. About 15% (*n* = 441) of the participants scored 21 or above, which may be indicative of symptom domains associated with BDD. There was a significant relationship between participants' gender and scoring above/below 21. Female participants were more likely than male participants to score 21 or above, χ^2^ (2, *N* = 437) = 60.60, *p* < 0.001, indicating that they were more at risk. Analyses by country showed that values above the cutoff point on the AAI registered the highest percentage of participants in Italy (18.1%), followed by several countries registering similar values (Table 5).

**Table 5:** Appearance Anxiety Inventory (AAI) and Self-Compassion Scale (SCS) results per country.

	**UK**	**Italy**	**Spain**	**Hungary**	**Portugal**	**Japan**	**Lithuania**	**Country differences**
AAI (scores ≥21) *n* = 2,873	45 (12.8%)	214 (18.1%)	39 (8.6%)	15 (15.2%)	51 (16.7%)	29 (16.9%)	48 (14.5%)	χ^2^ = 25.53 *p* < 0.001
SCS (scores <27) *n* = 2,785	81 (23.9%)	278 (24.3%)	62 (13.9%)	14 (14.9%)	32 (10.8%)	19 (11.2%)	39 (12.2%)	χ^2^ = 64.52 *p* < 0.001

*χ*^2^, *chi-square test*.

The sample's mean score on the SCS was 31.43 (*SD* = 5.71), with male participants (*M* = 32.35; *SD* = 5.25) showing significantly higher values than female participants (*M* = 30.92; *SD* = 5.89), *t*_(2784)_ = 6.55, *p* < 0.001, *d* = 0.26. The percentage of participants scoring below the cutoff point (i.e., values <27) was 16.6% (*n* = 525). The chi-square test showed that there was a significant association between gender and scoring above/below 27. Female participants were more likely than their male counterparts to score below 27, χ^2^ (2, *N* = 523) = 29.13, *p* < 0.001. The countries with the largest percentages of participants scoring below the cutoff point were Italy and the UK. These two countries registered similar percentages of low scorers (respectively, 24.3 and 23.9%) and greater percentages than the remaining countries (Table 5).

### Predictors of Physical Exercise and of Image and Performance-Enhancing Drugs (IPEDs) Use

Logistic regression on physical exercise [classified as 0, “no practice,” or 1, “practice,” according to the question, “Do you practice any sport(s)?”] included IPEDs consumption (0, “not used”; 1, “used”), AAI scores (0, <21; 1, ≥21), SCS scores (0, <27; 1, ≥27), and age in the model (Table 6). The strongest predictor of physical exercise was IPEDs use, with an odds ratio of 2.507, 95% CI 1.824–3.445, *p* < 0.001. The probability of practicing exercise almost tripled when participants used IPEDs compared to when they did not use them. Age was also significant and was positively related with physical exercise [odds ratio (OR) = 1.014, 95% CI 1.003–1.025, *p* = 0.012]. Appearance anxiety and self-compassion were non-significant predictors of physical exercise.

**Table 6:** Physical exercise logistic regression model (total and by gender).

		***B***	**ES**	**Wald**	**df**	***p***	**Odds ratio (OR)**	**Confidence interval (CI)**	
								**Min**	**Max**
Model I: physical	AAI (scores ≥21)	−0.280	0.164	2.921	1	0.087	0.756	0.548	1.042
exercise (total	SCS (scores <27)	0.225	0.152	2.193	1	0.139	1.252	0.930	1.687
sample) *N* = 1,995	IPEDs	0.919	0.162	32.122	1	0.000	2.507	1.824	3.445
	Age	0.014	0.005	6.251	1	0.012	1.014	1.003	1.025
	Constant	0.822	0.429	3.663	1	0.056	2.275		
Model II: physical	AAI (scores ≥21)	−0.108	0.418	0.066	1	0.797	0.898	0.395	2.038
exercise (men)	SCS (scores <27)	0.377	0.318	1.409	1	0.235	1.458	0.782	2.719
*N* = 564	IPEDs	1.427	0.328	18.950	1	0.000	4.165	2.191	7.917
	Age	0.025	0.010	6.348	1	0.012	1.026	1.006	1.046
	Constant	−0.142	0.865	0.027	1	0.870	0.868		
Model III: physical	AAI (scores ≥21)	−0.315	0.182	2.988	1	0.084	0.730	0.511	1.043
exercise (women)	SCS (scores <27)	0.183	0.174	1.109	1	0.292	1.201	0.854	1.688
*N* = 1,421	IPEDs	0.720	0.188	14.641	1	0.000	2.054	1.421	2.969
	Age	0.008	0.007	1.583	1	0.208	1.008	0.995	1.022
	Constant	1.145	0.502	5.197	1	0.023	3.141		

*Note: Physical exercise (0, “no practice”; 1, “practice”); IPEDs (0, “not used”; 1, “used”); AAI (0, scores <21; 1, scores ≥21), SCS (0, scores <27; 1, scores ≥27)*.

*AAI, Appearance Anxiety Inventory; IPEDs, image- and performance-enhancing drug; SCS, Self-Compassion Scale*.

Among male participants, the two significant predictors of physical exercising were IPEDs consumption (OR = 4.165, 95% CI 2.191–7.917, *p* < 0.001) and age (OR = 1.026, 95% CI 1.006–1.046, *p* = 0.012). IPEDs consumption was a strong positive predictor. All else being constant, men who use IPEDs were over four times more likely to practice exercise than not practice it. Among female participants, IPEDs use was also a significant predictor of exercising (OR = 2.054, 95% CI 1.421–2.969, *p* < 0.001). However, unlike male participants, age was not a significant predictor of physical exercise among female respondents. Appearance anxiety and self-compassion were not significant predictors of physical exercise among both male and female participants (Table 6).

The logistic regression on IPEDs consumption (classified as 0, “not used,” or 1, “used,” according to the question, “Have you taken supplements/products to reach your fitness goal/physical appearance during self-isolation?”) included problematic exercise (0, scores <24; 1, scores ≥24), AAI scores (0, <21; 1, ≥21), SCS scores (0, <27; 1, ≥27), and age (Table 7). The results showed that the strongest predictor of IPEDs use was problematic exercise (OR = 2.726, 95% CI 1.843–4.030; *p* < 0.001), followed by appearance anxiety (OR = 1.443, 95% CI 1.125–1.850, *p* = 0.004). Thus, the probability of using IPEDs was almost triple for those scoring 24 or above the cutoff point of 24 on the EAI, and almost one and a half times greater for those who scored on or above the cutoff point of 21 on the AAI, than for those who scored below the cutoff points. Like in the previous regression, self-compassion was statistically non-significant. However, unlike in the previous regression, age was also a non-significant factor here.

**Table 7:** Use of IPEDs logistic regression model (total and by gender).

		**B**	**ES**	**Wald**	**df**	***p***	**Odds ratio (OR)**	**Confidence interval (CI)**	
								**Min**	**Max**
Model I: IPEDs use (total sample)	AAI (scores ≥21)	0.366	0.127	8.330	1	0.004	1.443	1.125	1.850
*N* = 2,468	SCS (scores <27)	−0.047	0.118	0.160	1	0.689	0.954	0.757	1.202
	EAI (scores ≥24)	1.003	0.200	25.247	1	0.000	2.726	1.843	4.030
	Age	0.004	0.004	1.294	1	0.255	1.004	0.997	1.012
	Constant	−2.391	0.392	37.290	1	0.000	0.092		
Model II: IPEDs use (men) *N* = 843	AAI (scores ≥21)	0.648	0.261	6.160	1	0.013	1.912	1.146	3.189
	SCS (scores <27)	−0.092	0.212	0.187	1	0.665	0.912	0.602	1.382
	EAI (scores ≥24)	0.801	0.309	6.699	1	0.010	2.227	1.215	4.084
	Age	−0.007	0.006	1.555	1	0.212	0.993	0.982	1.004
	Constant	−1.668	0.638	6.833	1	0.009	0.189		
Model III: IPEDs use (women) *N* = 1,613	AAI (scores ≥21)	0.413	0.152	7.379	1	0.007	1.511	1.122	2.035
	SCS (scores <27)	−0.078	0.145	0.286	1	0.593	0.925	0.697	1.229
	EAI (scores ≥24)	1.100	0.267	17.019	1	0.000	3.003	1.781	5.063
	Age	0.013	0.005	6.176	1	0.013	1.013	1.003	1.023
	Constant	−2.961	0.512	33.512	1	0.000	0.052		

*IPEDs use (0, not used; 1, used); problematic exercise (0, scores <24; 1, scores ≥24); AAI (0, scores <21; 1, scores ≥21); SCS (0, scores <27; 1, scores ≥27)*.

*AAI, Appearance Anxiety Inventory; IPEDs, image- and performance-enhancing drug; SCS, Self-Compassion Scale*.

When only male participants were considered, the results were similar to those obtained for the whole sample. Problematic exercise was the strongest predictor of IPEDs use (OR = 2.227, 95% CI 1.215–4.084, *p* = 0.010), followed by appearance anxiety (OR = 1.912, 95% CI 1.146–3.189, *p* = 0.013), and both variables were positively associated with IPEDs use (Table 7). This suggests that male participants who scored above the cutoff points (in both instruments) had about double the probability to use IPEDs than male participants who scored below the cutoff points. When only female respondents were considered, again, the strongest predictor of IPEDs use was problematic exercise (OR = 3.003, 95% CI 1.781–5.063, *p* < 0.001), followed by appearance anxiety (OR = 1.511; 95% CI 1.122–2.035, *p* = 0.007), and both variables were positively associated with IPEDs use as well (Table 7). Additionally, age was significant, though only for female participants (OR = 1.013; 95% CI 1.003–1.023; *p* = 0.014). This indicates that problematic exercise was a strong predictor among female respondents, increasing by three times their probability of using IPEDs. This probability also increased with appearance anxiety and with age, though to a lesser extent (Table 7).

### Predictors of Change in Physical Exercise, in the Use of Image and Performance-Enhancing Drugs (IPEDs), and in Mental Health State During the Self-Isolation Period

To assess changes during the self-isolation period, logistic regressions were conducted on three questions. One question assessed changes in physical exercise: Whether participants had a significant change in their fitness routine during this self-isolation period (0, “no”; 1, “yes”). Another assessed changes in their use of IPEDs (0, “never used”; 1, “never used, but started during isolation”). The third assessed changes in their mental health state, and only participants who had responded “yes” to the question on whether they had a mental health diagnosis were included: Whether this self-isolation period worsened their psychological discomfort (0, “no”; 1, “yes”). The results of the regressions are presented in Table 8, respectively, on (1) change in physical exercise, (2) change in the use of IPEDs, and (3) change in mental health. The same predictors as before were included in the regression models. To inspect whether changes in physical exercise, use of IPEDs, and mental health were associated with being a key worker, the latter aspect was additionally included in the models (0, “non-key worker”; 1, “key worker”).

**Table 8:** Logistic regression on change in physical exercise, in IPEDs use, and in mental health.

		**B**	**ES**	**Wald**	**df**	***P***	**Odds ratio (OR)**	**Confidence interval (CI)**	
								**Min**	**Max**
Model I: Change in physical exercise Have you had a significant change in	AAI (scores ≥21)	0.234	0.153	2.355	1	0.125	1.264	0.937	1.704
your fitness routine during this self-isolation period? 0, “no”; 1, “yes”	SCS (scores <27)	−0.045	0.132	0.116	1	0.733	0.956	0.738	1.239
*N* = 2,464	IPEDs	0.283	0.110	6.633	1	0.010	1.327	1.070	1.645
	Gender	−0.114	0.105	1.194	1	0.275	0.892	0.727	1.095
	Age	0.002	0.004	0.227	1	0.634	1.002	0.994	1.011
	Key worker (No/Yes)	−0.321	0.103	9.678	1	0.002	0.725	0.592	0.888
	Constant	1.189	0.410	8.408	1	0.004	3.283		
Model II: Change in IPEDs use Have you taken more	AAI (scores ≥21)	0.060	0.252	0.057	1	0.812	1.062	0.648	1.739
supplements/products to reach your fitness goal/physical appearance	SCS (scores <27)	−0.204	0.218	0.877	1	0.349	0.815	0.532	1.250
during self-isolation 0, “never used”; 1, “never used, but started during	EAI (scores ≥24)	0.821	0.360	5.184	1	0.023	2.272	1.121	4.606
isolation”	Gender	−0.198	0.179	1.220	1	0.269	0.820	0.577	1.166
*N* = 1,917	Age	0.000	0.007	0.004	1	0.951	1.000	0.985	1.014
	Key worker (No/Yes)	−0.068	0.186	0.134	1	0.714	0.934	0.648	1.346
	Constant	−2.613	0.789	10.975	1	0.001	0.073		
Model III: Change in mental health Has this self-isolation period	AAI (scores ≥21)	0.648	0.236	7.514	1	0.006	1.912	1.203	3.039
worsened your psychological discomfort 0, “no”; 1, “yes”	SCS (scores <27)	−0.674	0.216	9.722	1	0.002	0.510	0.334	0.779
*N* = 438	EAI (scores ≥24)	0.271	0.413	0.428	1	0.513	1.311	0.583	2.947
	IPEDs	0.0072	0.224	0.104	1	0.748	1.075	0.693	1.665
	Gender	0.277	0.262	1.119	1	0.290	1.319	0.790	2.203
	Age	−0.005	0.010	0.209	1	0.648	0.995	0.975	1.016
	Key worker (No/Yes)	−0.018	0.250	0.005	1	0.943	0.982	0.602	1.604
	Constant	−0.992	0.905	1.202	1	0.273	0.371		

*Note: AAI (0, scores <21; 1, scores ≥21); SCS (0, scores <27; 1, scores ≥27); IPEDs use (0, not used; 1, used); EAI (0, scores <24; 1, scores ≥24); Gender (0, men; 1, women); key worker (0, “non-key worker”; 1, “key worker”)*.

*AAI, Appearance Anxiety Inventory; IPEDs, image- and performance-enhancing drug; SCS, Self-Compassion Scale*.

Significant aspects associated with the change in physical exercise were IPEDs consumption (OR = 1.327, 95% CI 1.070–1.645, *p* = 0.010) and being a key worker (OR = 0.725, 95% CI 0.592–0.888, *p* = 0.002). This means that changes in practicing exercise were more likely when participants used IPEDs and were non-key workers.

The only predictor of change in IPEDs consumption was scoring 24 or above the cutoff of 24 on the EAI (OR = 2.272, 95% CI 1.121–4.606, *p* = 0.006). This was a strong predictor, reflecting the idea that initiating IPEDs use during self-isolation was about two times more likely when participants scored above the cutoff point for problematic exercise.

Change in mental health was significantly and positively associated with anxiety about appearance (OR = 1.912, 95% CI 1.203–3.039, *p* = 0.002) and negatively associated with self-compassion (OR = 0.510, 95% CI 0.334–0.779, *p* = 0.002). This means that, all else held constant, discomfort during the confinement period among participants with mental health history was more likely to worsen with increased anxiety about appearance and decreased self-compassion.

## Discussion

This study sought mainly to (1) characterize the practice of physical exercise and the consumption of IPEDs in a sample of the general population from seven countries worldwide during the lockdown and other restrictive measures, (2) analyze the potential associations of these behaviors with appearance anxiety (and the risk of BDD) and with self-compassion as a possible buffer of negative effects, and (3) analyze changes in those behaviors and in psychological discomfort during the lockdown and associated factors.

Scores of 24 or above such a cutoff score on the EAI are indicative of problematic exercising and are suggestive of exposure to the adverse effects of exercise, namely, injuries [e.g., (24, 73)]. Excessive exercise also negatively impacting well-being and becoming harmful (28, 29). Although studies in this area are recent and scarce, the percentage of respondents at risk of problematic exercising in our sample (4.3%) was large, comparing with the proportions found in other studies also conduced within community samples but prior to the COVID-19 pandemic [e.g., reported percentages range between 0.3 and 0.5% (24)]. This percentage was smaller than the proportion found among gym users before the COVID-19 pandemic (11.7%) (23) or among the exercise practitioners of Spanish-speaking countries (15.2%) in the recent study that was carried out during the COVID-19 lockdown period—in April of 2020 (65). Our results further showed that more male than female respondents displayed a risk of problematic exercising, which is consistent with previous studies [e.g., (23, 42–44)].

The multicultural nature of our study made it possible to identify a significant association between problematic exercise and country. The UK registered the largest percentage of participants at risk for problematic exercising (11.0%). This was more than double the value found in Spain (5.4%) and generally more than triple the values registered in the remaining countries that ranged from 2.4% (in Lithuania) to 3.7% (in Hungary). The percentage in the UK was similar to that previously reported among gym users (23); this may be explained by the fact that the UK also had a large percentage of participants who practice exercise (97.1%; Table 1), greater than five of the other countries. However, Hungary had a larger number of participants who practice exercise (98.2%; Table 1), yet showed a much smaller percentage of respondents at risk for problematic exercising (3.7%) than the UK (11.0%) or Spain (5.4%). The remaining three countries (Portugal, Italy, and Lithuania) displayed the largest percentages of participants who did not practice exercise with scores ranging between 15% and 20% (Table 1), and their respective percentages of participants at risk for problematic exercising (ranging between 2.4% for Lithuania and 3.1% for Italy) were closer to Japan's than to their European counterparts (i.e., the UK and Spain). It is possible that cross-cultural differences play a role in determining the rationale behind the practice of physical exercise (74). Such a hypothesis is supported by the fact that even within fitness settings, where risk of problematic exercising is higher, a larger percentage of problematic exercisers was found in the UK (16.1%) compared to that in Hungary (9.3%) or Italy (7.9%) in a study that was carried out just before the COVID-19 pandemic (23). In Spain, high scores of problematic exercising were found in the pre-COVID-19 period among university students (6%), although a different measurement was used [Exercise Dependence Scale (EDS-R)] (75). Another study found an even greater percentage (14.9%) of students at risk of addiction on the EAI (76). The prevalence of problematic exercising in the general Spanish population in other studies was about 3% (74).

Although the concept of EA is not consensual, the comparatively high number of participants displaying such risk among the general population during the COVID-19 lockdown period suggests that results emerging from our study should be taken into consideration and inform future restrictive measures, while emphasizing group vulnerabilities in cross-cultural differences.

The same argument is valid for IPEDs consumption. Overall, 28% of the respondents across the general population used these products during the lockdown. Among these, 6.4% began a new use of IPEDs, while only 1.5% reduced their use. This might suggest that the use of IPEDs is a coping strategy to deal with the challenges posed by the COVID-19 pandemic, including distress related to body image experienced during quarantine. As previously suggested (50), the social pressure to achieve a “perfect body,” combined with the easy online access to IPEDs supported by aggressive and misleading advertisement strategies, promising fast solutions, could be a possible explanation for the observed growing phenomenon during the lockdown. Further, participants in our study mostly boosted their image and performance with the consumption of legal compounds (e.g., medicine, supplements), which in 43.2% of the cases were acquired from the Internet possibly with no professional advice and supervision as previously noticed (29).

Male respondents reported using IPEDs significantly more than female respondents, and their intake significantly differed across countries, with Hungary and Japan displaying the largest consumption (Table 3). In the case of Hungary, this might have been influenced by the fact that the sample had the lowest representation of students (9%) and, consequently, had the highest mean age, which could be a contributing factor to such difference with other countries. Further, Hungarians recorded the smallest percentage of participants who did not exercise (1.8%) and, compared to the others, their sample was composed of the highest proportion of both runners (27.9%) and swimmers (28.8%). Based on such characteristics, it appears that either by chance, or due to self-selection, the Hungarian population was the most physically active population within our sample, thus explaining the results concerning the IPEDs use.

Considering that, so far, the widespread consumption of IPEDs has been associated only with professional fitness settings (23), the results from our study across the general population are surprisingly high compared with those emerged from previous studies (23). Interestingly, the countries registering the greatest percentages of IPEDs consumers were also those presenting the greatest percentages of participants practicing exercise with the exception of Lithuania (Table 1). In this latter case, the smallest percentage of participants at risk of EA across the entire sample was also reported (Table 2). Overall, our results are indicative that IPEDs are also used outside the context of problematic or excessive exercise and, especially in the case of Lithuania, outside the context of the practice of exercise. Nevertheless, excessive levels of exercise have been suggested to be associated with a greater tendency for using IPEDs (23), indicating that careful attention should be given to excessive supplement use among individuals engaging in problematic levels of exercise practice.

Finally, those most affected by appearance anxiety were in Italy (18.1%) and Japan (16.9%), mainly female participants. Although the literature is limited, a previous study indicated that Italian women compared with South Asians and British women seemed to consider themselves more “overweight” and “fairly unhappy with the way they look” (77). Another study also indicated higher levels of thin-ideal internalization and peer and media pressure across Italian women (78). Such an attitude might have been magnified by stricter national lockdown at the time of the data collection compared to other countries. Italy was in fact the first country in the European Union to be affected by the pandemic (79), causing an unprecedented negative impact on the mental well-being and significant emotional distress that could have reinforced the high scores on the AAI in our study.

At the same time, young Japanese females have been shown to have a stronger desire to get slender bodies, manifested by the feelings of ideal body images in individuals with lower body mass index (BMI) compared to Europeans (80). This difference in “ideal body image” among the countries could explain the higher rate of Japanese population with high AAI scores.

Our regression models showed a strong relation between physical exercise and IPEDs use. Using IPEDs significantly predicted the probability of practicing exercise in the whole sample, particularly among males (for whom the probability increased by four and a half times). An unexpected result was that self-compassion was non-significantly associated with practicing exercise.

Our regressions on the use of IPEDs additionally showed that the risk of addiction to exercise (i.e., scores ≥24 on the EAI) significantly predicted IPEDs use across the three considered groups and note in the three groups considered (whole sample and male and female participants), doubling or tripling the probability of consumption. As expected, high anxiety about physical appearance also significantly increased the probability of using IPEDs in the three groups (by at least one and a half to two times more), underscoring the role of psychological discomfort on the consumption of these products. However, again, self-compassion was non-significantly associated with using IPEDs.

In sum, consumption of IPEDs predicted the practice of physical exercise, thus the risk of problematic exercising predicted IPEDs consumption. These results support those in a recent study reporting, for the first time, the association between the consumption of IPEDs and problematic exercising (23). High appearance anxiety predicted more consumption of IPEDs but not the practice of exercise. Vulnerable groups are thus particularly likely to use IPEDs. This is consistent with the easy access to IPEDs and the notion that advertising strategies promising easy and quick results from their consumption might be transforming such consumption into a preferred strategy compared to exercising, particularly during the period in which its practice has become more difficult due to restrictive measures and possible lack of space at home.

Regarding changes in habits during the COVID-19 lockdown period, a small proportion of the sample participants reported having started using IPEDs during isolation (6.4%). Changes in fitness routines (though not in the use of IPEDs) were significantly less likely to occur if participants were key workers rather than non-key workers. Seemingly, key workers were able to maintain the various domains of their lives functioning during the lockdown. The fact that IPEDs use was one aspect that significantly contributed to the changes in exercise practice and that the risk of problematic exercising was the only aspect that significantly contributed to the increase in the use of IPEDs during the quarantine period underlines the potential influence that the particular circumstances of restriction might have in exacerbating these phenomena and the association between them.

Several participants with mental health conditions considered that their psychological discomfort has worsened during isolation (47.9%) (Table 1). Both increased symptom domains associated with BDD and decreased self-compassion contributed significantly to this change. Such changes in mental health, related to body image (dis)satisfaction and with difficulties in emotional self-regulation, could contribute to alter behaviors and habits that, although intended to minimize or supress the dissatisfaction, could become harmful to vulnerable groups, affecting several life dimensions. In this study, however, we found no evidence for the occurrence of these altered behaviors in the sequence of worsening psychological discomfort because the association of these changes in mental health with both the risk of problematic exercising and the use of IPEDs was statistically non-significant.

Regarding age, there was a positive association between the age of the participants and exercising in the total sample but, also between the age of the participants and consumption of IPEDs among female respondents. According to Szabo (28, 49), adherence to a healthier lifestyle should increase with age, and it is necessary to understand the effect of this variable on exercising and the type of IPEDs used to better understand this association.

Overall, our culturally enriched investigation was a timely contribution to a better understanding of the risks, and not only the benefits, associated with exercise and the IPEDs consumption as coping strategies during a period of highly restrictive measures of freedom and social contact due to the COVID-19 pandemic. Despite the adversities faced by all of us, we were able to rapidly capture a large set of data from a cross-cultural sample in seven countries worldwide right at the beginning of the pandemic, which reflected significant changes in variables of interest. However, our effort is not exempt from limitations. First, the study is based on a non-stratified sample and on voluntary participation, which might introduce selection bias because the representativeness of the population is not ensured. Second, the snowball recruitment process and the online nature of data collection might help explain some of the high prevalences obtained. Nevertheless, the studies on this topic were based on similarly voluntary participation, making comparisons possible. Third, the study relies on self-reported measures, thus it is subject to the biases that characterize this modality of inquiry, namely, regarding consumption of IPEDs, which was not validated through any biological testing. Fourth, clinical interpretations of the results require caution because, for some instruments (notably the AAI), a statistical cutoff point was used to identify high anxiety about physical appearance and the symptom domains associated with BDD, instead of a clinical cutoff point, which is not available. Fifth, information on the individual's history of exercise and consumption of IPEDs is lacking, to support a better understanding of the excessive and the problematic nature of such behaviors, including their frequency and duration. Sixth, the study design does not allow causal inferences, although conclusions on the relative associations between the variables were possible.

We are confident that future studies can further illuminate our findings by addressing and overcoming such limitations, which we were unable to control during this narrow window of opportunity. Additional evidence should be collected on the so far poorly understood relationships between physical exercise and the use of IPEDs and the role of precipitating and of protecting factors (i.e., problematic exercising and appearance anxiety as precipitating factors and self-compassion as a protecting factor) that very recent investigations, including this study, have started to show. The inclusion of psychiatric interviews and/or objective tests would also contribute to further validate the self-reported responses of our online sample. Having identified the most at risk population, more targeted and effective prevention strategies could be more easily be implemented, even more importantly during periods of personal confinement. Some reinforcement programs on addiction health care have been launched during the pandemic (81). They address behaviors other than exercise or the use of enhancement drugs, but similar initiatives could be created in the future, targeting the latter aspects as well. It is worth noting that those affected by problematic exercise and IPEDs use under normal conditions rarely come to the attention of health professionals in part because of the “normalization” of their behavior in society and the fact that they do not consider themselves “drug users” in a traditional sense. If care is sought, primary care doctors, as opposed to psychiatrists or psychologists, are consulted. By identifying those who are most affected, including frequent exercisers, public health and clinical interventions can be developed and more adequately help them to adjust better, thereby relieving personal distress, improving health and well-being, restoring family and occupational and social functioning, and ultimately supporting the economic growth of our countries.

## Data Availability Statement

The raw data supporting the conclusions of this article will be made available by the authors, without undue reservation.

## Ethics Statement

The studies involving human participants were reviewed and approved by Human Sciences Ethics Committee at the University of Hertfordshire (HSK/SF/UH/00104). The patients/participants provided their written informed consent to participate in this study.

## Author Contributions

Conceptualization: OC, PS, GM, and NF. Methodology: OC, PS, GM, NF, AD, and IC. Formal analysis: JB and AD. Investigation and data collection: ID, KÁ, AD, PS, RM, MG-M, AV, EA-A, RS-L, JB, IG-B, AP, ZD, AS, HF, MS, and KK. Resources: AD, OC, and NF. Data curation: OC, AD, JB, RM, and ZD. Writing-original draft preparation: AD. Writing-review and editing: IC, RM, JB, FB, and KI. Visualization: AD and IC. Supervision: OC, AD, and IC. Project administration: OC. Funding acquisition: NF, OC, and AD. All authors have read and agreed to the published version of the manuscript.

## Conflict of Interest

The authors declare that the research was conducted in the absence of any commercial or financial relationships that could be construed as a potential conflict of interest. The handling editor declared a shared affiliation with several of the authors MG-M, AV, and EA-A at time of review.
